# DNA methylation patterns contribute to changes of cellular differentiation pathways in leukocytes with LOY from patients with Alzheimer´s disease

**DOI:** 10.1007/s00018-025-05618-8

**Published:** 2025-02-25

**Authors:** Marcin Jąkalski, Bożena Bruhn-Olszewska, Edyta Rychlicka-Buniowska, Hanna Davies, Daniil Sarkisyan, Maciej Siedlar, Jarosław Baran, Kazimierz Węglarczyk, Janusz Jaszczynski, Janusz Ryś, Vilmantas Gedraitis, Natalia Filipowicz, Alicja Klich-Rączka, Lena Kilander, Martin Ingelsson, Jan P. Dumanski

**Affiliations:** 1 3P-Medicine Laboratory, Medical University of Gdańsk, Dębinki 7, 80-211, Gdańsk, Poland; 2 Department of Immunology, Genetics and Pathology and Science for Life Laboratory, Uppsala University, Uppsala, Sweden; 3 Department of Clinical Immunology, Institute of Paediatrics, Jagiellonian University, Collegium Medicum, Kraków, Poland; 4 Department of Urology, Maria Skłodowska-Curie National Research Institute of Oncology, Kraków, Poland; 5 Department of Tumor Pathology, Maria Skłodowska-Curie National Research Institute of Oncology, Kraków, Poland; 6 Department of Public Health and Caring Sciences/Geriatrics, Uppsala University, Uppsala, Sweden; 7 Department and Clinic of Internal Medicine and Gerontology, Jagiellonian University, Collegium Medicum, Kraków, Poland; 8 Krembil Brain Institute, University Health Network, Toronto, ON Canada; 9 Tanz Centre for Research in Neurodegenerative Diseases, Departments of Medicine and Laboratory Medicine & Pathobiology, University of Toronto, Toronto, ON Canada

**Keywords:** DNA methylation, CpG dinucleotide methylation, Loss of chromosome Y, Alzheimer’s disease, Gene expression regulation

## Abstract

Alzheimer’s disease (AD) is a common and increasing societal problem due to the extending human lifespan. In males, loss of chromosome Y (LOY) in leukocytes is strongly associated with AD. We studied here DNA methylation and RNA expression in sorted monocytes and granulocytes with and without LOY from male AD patients. Through multi-omic analysis, we identified new candidate genes along with those previously associated with AD. Global analyses of DNA methylation in samples with LOY vs. normal state showed that hypomethylation dominated both in granulocytes and monocytes. Our findings highlight LOY-related differences in DNA methylation that occur in gene regulatory regions. Specifically, we observed alterations in key genes involved in leukocyte differentiation: *FLI1*, involved in early hematopoiesis; *RUNX1*, essential for blood cell development; *RARA*, regulating gene expression in response to retinoic acid; *CANX*, crucial for protein folding; *CEBPB*, a transcription factor important for immune responses; and *MYADM*, implicated in cell adhesion and migration. Moreover, protein–protein interaction analysis in granulocytes identified that products of two of these genes, *CANX* and *CEBPB*, are key hub proteins. This research underscores the potential of multi-omic approach in pure hematopoietic cell populations to uncover the molecular underpinnings of AD. Finally, our results link previous analysis showing impact of LOY on leukocyte differentiation, LOY-associated transcriptional dysregulation and GWAS studies of LOY.

**Supplementary Information:**

The online version contains supplementary material available at 10.1007/s00018-025-05618-8.

## Introduction

Loss of chromosome Y (LOY) in leukocytes from aging males [1] is the most common post-zygotic mutation, detectable in whole blood DNA from > 40% of men above the age of 70 years [2], reaching 57% in the analysis of bulk DNA from 93-year-old men [3]. A single-cell analysis of Peripheral Blood Mononuclear Cells (PBMCs) derived from 29 aging men (median age 80 years) identified cells with LOY in every studied subject [4]. The presence of LOY has also been reported in other tissues although with lower frequencies [3, 5]. Importantly, a serial analysis of blood samples showed that LOY is a dynamic process [1, 6, 7].

Major risk factors for LOY include age, smoking, and germline predisposition as well as environmental and occupational hazards [1, 2, 8–14]. It has also been suggested that LOY affects various lineages of hematopoietic cells with different frequencies and that it plays a role in the dysregulation of autosomal genes through LOY-Associated Transcriptional Effects (LATE) in a pleiotropic manner [4]. Moreover, dysregulation of large sets of various immune genes was pronounced in LOY cells [4, 15–17].

LOY has been associated with increased risk for all-cause mortality as well as with chronic and acute age-related diseases inside and outside of the hematopoietic system [1, 5, 7, 18, 19], with causal effects of LOY already shown for cardiac fibrosis and bladder cancer [20–22]. Notably, a strong association between LOY and late-onset Alzheimer’s disease (LOAD), the most common neurodegenerative disorder was observed. Males with hematopoietic LOY had a 6.8-fold greater risk for Alzheimer’s disease (AD) diagnosis [9]. This effect is comparable to that of the strongest genetic risk factor for LOAD, the *ε*4 allele of the apolipoprotein E gene (*APOE*). The presence of one or two copies of *APOE ε*4 increases the risk of developing LOAD by a factor of 3 up to 15-fold in a dose-dependent manner [23, 24]. In addition to *APOE,* genome-wide association studies (GWAS) have identified several other AD risk genes [23, 25–27]. Whereas early onset AD (EOAD) sometimes can be explained by mutations in either of three genes (*APP, PSEN1, PSEN2*), LOAD is believed to be caused by complex genetics in combination with environmental factors [26].

The replication of the association between LOY and AD in additional cohorts, also applying a different methodological approach, has recently been shown in two reports [28, 29]. Transcriptome analyses further suggested that LOY might indeed play an important role in the pathogenesis of AD [15, 30]. Specifically, a recent study of LOY in human brain tissues from healthy aging subjects has shown that microglia have the highest percentage of LOY and remarkably, there was a significant increase of LOY in microglia from male AD donors [15]. Brain microglia and circulating monocytes represent functionally closely related cells, and monocytes from blood can migrate across the blood–brain barrier (BBB) in response to inflammatory stimuli in various diseases, including AD [31–34]. The process of clearance of amyloid plaques by microglia in the brain and clearance of circulating amyloid-beta from blood by monocytes has further been suggested as an important disease mechanism in AD [35, 36]. Moreover, apart from the impact of monocytes in AD pathogenesis, neutrophils, the most abundant granulocyte cells, exhibit a hyperactive phenotype by elevated production of reactive oxygen species (ROS) in AD, leading to inflammation and disease progression [37].

Multiple studies have also shown that epigenetic processes are often dysregulated and might play a role in the development and progression of AD. DNA methylation at CpG sites provides a stable epigenetic modification that usually silences (alternatively promotes in case of demethylation) the transcription of adjacent gene(s) [38, 39]. Altered DNA methylation levels in whole blood DNA were identified as associated with worse cognitive performance and accelerated rate of AD progression [40–43]. Furthermore, covalent modifications of histones, and higher-level three-dimensional structures (such as topologically associating domains, TADs), are other examples of dynamic epigenetic regulators. TADs constitute one of the forms of chromosomal organization within a nucleus into functional compartments [44]. Genes bound by the same TAD are usually regulated in a coordinated manner, as TADs facilitate interactions between the genes and their distant regulatory elements. DNA methylation and histone modifications, such as histone methylation and acetylation, are in a constant interplay [45, 46]. Recent studies showed that epigenetic processes are distorted in AD patients and that the epigenetic control of enhancers may have an important pathogenetic role [46–48]. Of note is also here that LOY leads to complete inactivation of *KDM5D* and *UTY/KDM6C* genes, which are histone demethylases and are located on the male-specific part of chromosome Y. It has been shown that reduced expression of KDM5D results in higher H3K4me3 levels at the target gene promoter [49]. Moreover, disruption of *UTY/KDM6C* enhances chromatin accessibility for genes involved in various cellular processes in cardiac macrophages [50]. The above reasoning provides a motivation and rationale for this study.

We hypothesized that LOY may be associated with global changes in DNA methylation and may lead to the discovery of new, specific candidate genes. We have taken a novel approach to study AD by incorporating multi-omics data (DNA methylation and gene expression) and the LOY status of the studied individuals, to delineate candidate genes that might be involved in the pathogenesis of this condition. For this purpose, we took advantage of pure flow cytometry-sorted populations of granulocytes and monocytes with or without LOY.

## Materials and methods

### Study group

Blood samples from patients diagnosed with AD were collected from male subjects in Uppsala, Sweden and Kraków, Poland for the purpose of estimating LOY levels as described [4]. The availability of sufficient amount of DNA was used to select a group of 43 individuals for complementing the previous study with DNA methylation analyses. Samples from AD patients were collected during January 2015 to May 2018, at the Geriatric/Memory Clinic, Uppsala Academic Hospital Sweden. Additional samples from AD patients were collected from January 2017 to May 2018 at the Clinic of Internal Medicine and Gerontology of the Jagiellonian University in Kraków. The criteria for recruitment of AD patients were ongoing clinically and radiologically confirmed diagnosis, intermediate or severely advanced disease.

The study was approved by the local research ethics committee in Uppsala, Sweden (Regionala Etikprövningsnämnden i Uppsala (EPN): Dnr 2005-244, Ö48–2005; Dnr 2013/350; Dnr 2015/092; Dnr 2015/458; Dnr 2015/458/2, the latter with update from 2018) and the Bioethical Committee of the Regional Medical Chamber in Kraków, Poland (No. 6/KBL/OIL/2014). All participants or their next of kin have given their written informed consent to participate. The study was conducted according to the guidelines of the Declaration of Helsinki.

### Sample preparation

We collected 16 ml of blood into two BD Vacutainer® CPT™ Mononuclear Cell Preparation Tubes (BD). Next, peripheral blood mononuclear cells (PBMCs) were isolated following the manufacturer’s instructions. The PBMCs were then washed with PBS. Additionally, we collected 16 ml of whole blood into two BD Vacutainer® K2 EDTA tubes (BD). Red blood cells were lysed using 1 × BD Pharm Lyse™ lysing solution (BD). Isolated white blood cells (WBCs) were washed with PBS. Subsequently, targeted cell populations were immediately sorted from the isolated PBMCs and WBCs using FACS [4]. In brief, live cells were sorted based on their FSC and SSC. Monocytes were defined based on their size and CD14+ signature, whereas granulocytes were defined based on their size and granularity. The cells were sorted to ensure a purity of over 96%. After sorting, the cell fractions were separated for subsequent DNA or RNA extraction. The cells intended for RNA extraction were dissolved with RNAprotect Cell Reagent (Qiagen). All cell fractions were pelleted and frozen at −70 °C for downstream analysis.

### Estimation of LOY levels

DNA was extracted and quantified from each isolated cell population following established protocol [4]. DNA was genotyped using three different versions of SNP-arrays InfiniumCoreExome-24v1-1, InfiniumOmniExpressExome-8v1-3 and InfiniumQCArray-24v1 (Illumina). All genotyping experiments were performed following the manufacturer’s instructions at the Science for Life technology platform SNP&SEQ at Uppsala University, Sweden. All included experiments passed strict quality control at the genotyping facility: the SNP call rate for all samples was > 98%, and the LogRdev value was < 0.2. The results from Illumina SNP arrays consist of two main data tracks: log R ratio (LRR) and B-allele frequency (BAF). We analyzed Illumina output files by using Nexus Copy Number version 5.1 (BioDiscovery, CA, USA), which applies a “Rank Segmentation” algorithm based on the circular binary segmentation (CBS) approach [51]. Additional QC criteria as well as calculation of mLRRY were done as described [4]. The percentage of cells with LOY (%LOY) in each sample was estimated using the published formula, i.e., 100*(1−(2^(2*mLRRY)^)) [6].

### Bisulfite conversion of DNA samples

Based on the DNA concentrations determined by Quant-iT PicoGreen dsDNA Assay (Thermo Scientific), 250 ng of each DNA sample were used in bisulfite conversion of methylated CpG sites using the EZ DNA MethylationTM Kit (Zymo Research). The bisulfite converted DNA was eluted and used for methylation analysis according to the manufacturer’s protocol.

### Methylation analysis

Methylation profiling was performed with the Infinium assay using the MethylationEPIC_v-1-0 array (Illumina) according to the manufacturer’s protocol. The scanning of the EPIC arrays and determination of signal intensities were performed by the iScan System (Illumina). Intensities were normalized using Illumina’s internal normalization probes and algorithms, with background subtraction.

### Analysis of DNA methylation data

The raw IDAT files were read into R using the readEPIC function from the wateRmelon package. Next, lumiMetyC (with quantile normalization) and BMIQ functions from the lumi and wateRmelon packages respectively were used to perform data normalization. The data was filtered by minimum detection p-value and all probes overlapping with known SNPs were removed. Probe annotation was performed within R using the package *IlluminaHumanMethylationEPICanno.ilm10b4.hg19*. Here we focused on several different annotation categories, such as *UCSC_RefGene_NAME, Relation_to_Island* (OpenSea, Island, N_Shore, N_Shelf, S_Shore, S_Shelf), *Regulatory_Feature_Group* (Promoter_Associated, Gene_Associated, NonGene_Associated, Unclassified), *UCSC_RefGene_Group* (TSS1500, TSS200, 5’UTR, 1stExon, Body, ExonBnd, 3′UTR).

To identify statistically significant differences in methylation between the compared groups we used the *dmpFinder* function from the *minfi* package. Probes located on chromosome Y were removed prior to the analysis. Significant differentially methylated probes (DMPs) were called when absolute average change in methylation between sample groups was at least 0.1 (M-value) and Benjamini-Hochberg (BH) adjusted P-value < 0.05. To intersect DMPs with gene regions, we used the UCSC based annotations and the promoter regions of UCSC genes were defined by a genomic window of ± 2 kb from TSS using the *promoters* function from the GenomicRanges R package [52]. Visualization of genomic neighborhood was performed using *Gviz* package. Gene annotations were plotted for *hg19* version of the human genome using UCSC-based gene annotations.

### Bulk RNA-seq data processing and reanalysis

RNA-seq data of sorted cell populations were obtained from our previous study. We selected only RNA samples from Alzheimer’s disease (AD) patients and for which genomic DNA was available [4]. The sequenced AmpliSeq data were basecalled with the Ion Torrent Suite Sever 5.8.0.RC2 software (Thermo Fisher). The generated reads were aligned to the human transcriptome reference (hg19 AmpliSeq Transcriptome ERCC v1) with TMAP mapper. Next, the raw gene expression counts for all amplicon targets in the assay (20,183) were merged to create separate count matrices for each of the two cell types (granulocytes and monocytes). Count data were processed with the R library *edgeR* version 3.28.1 [53]. We kept only the genes with expression levels above 1 count per million in at least six samples to remove low quality data. Further assessment of data quality using principal component plots revealed sample grouping corresponding to sequencing batch and patient source. The batch effects were thus adjusted using the *ComBat_seq* function from the *sva* package (version 3.35.2) [54]. Genewise Negative Binomial Generalized Linear model was applied to test for differential expression of genes between the AD patients with and without LOY. We considered genes to be significantly differentially expressed by applying a threshold of < 0.05 to the corrected p-values (FDR, Benjamini–Hochberg adjustment).

### Annotation of genes, gene ontologies and KEGG pathways

Gene set enrichment analyses were performed in R using the *Cluster Profiler* package. Gene symbols (either DMG or DEG) were mapped to Entrez ID. As a background for enrichment analyses for DEGs we used a set of all genes expressed in each tissue (based on bulk RNA-seq data). DMGs were tested against the background of all genes.

### Analyses of protein–protein interaction networks using STRING

We used the STRING database to identify if any protein–protein interaction networks existed among the genes identified in our study. Here we decided to utilize only genes showing alterations of DNA methylation within their promoter region and following the canonical model of DNA methylation vs. expression change. Thus, we applied 157 and 10 genes from granulocytes and monocytes, respectively, to the search box “multiple proteins” in the STRING database server (https://string-db.org/). Disconnected nodes, as well as nodes with less than 3 connections were removed [55].

### Analyses of transcription factor binding site enrichment

We used SEA (Simple Enrichment Analysis) from the MEME package to identify motifs that are relatively enriched among the promoter regions (± 2 kb from transcription start site) of genes that are both differentially methylated and differentially expressed [56]. A search for enriched motifs was performed against promoter regions of non-differentially methylated genes using the HOCOMOCO database of transcription factor binding motifs (version 11, core HUMAN collection, 401 motifs) [57]. Due to the limitations of the MEME online server, the analyses were run locally using the stand-alone version of the MEME suite (version 5.5.5).

## Results

### Study subjects and measurements of LOY

**Fig. 1:**
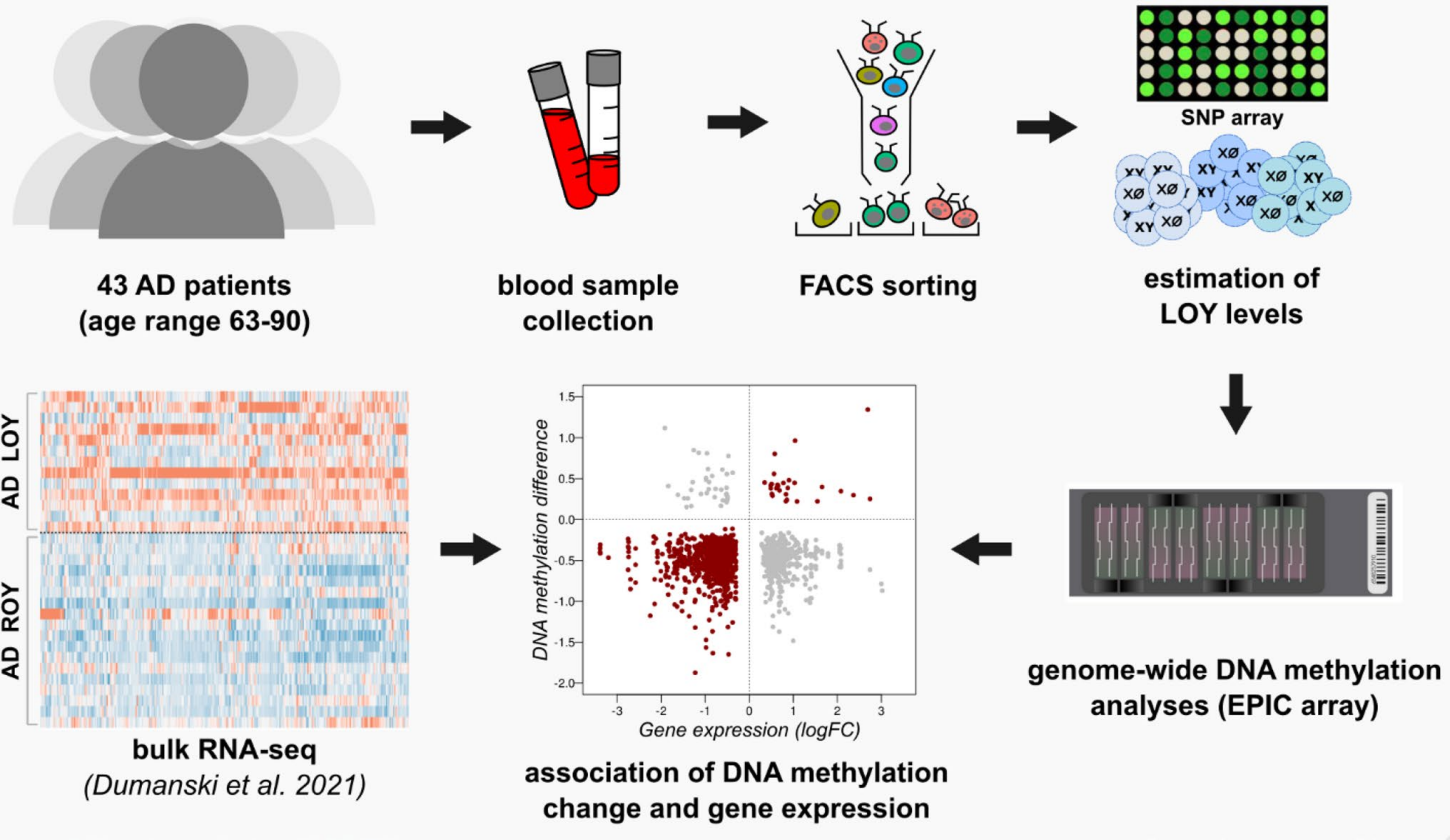
Graphical abstract of the study. We selected 43 AD patients and collected their blood. Leukocytes were then sorted on FACS for two main groups of cells, i.e. granulocytes and monocytes. Estimation of %LOY in each sample (% of cells without Y chromosome) was done using SNP array. ROY stands for Retention Of chromosome Y group of samples. In parallel, samples were subjected to genome-wide DNA methylation profiling using the Illumina EPIC Beadchip. We further incorporated bulk RNA-seq data from our previous project for the correlation of variation in DNA methylation with the gene expression levels in samples from the same patients

**Fig. 2:**
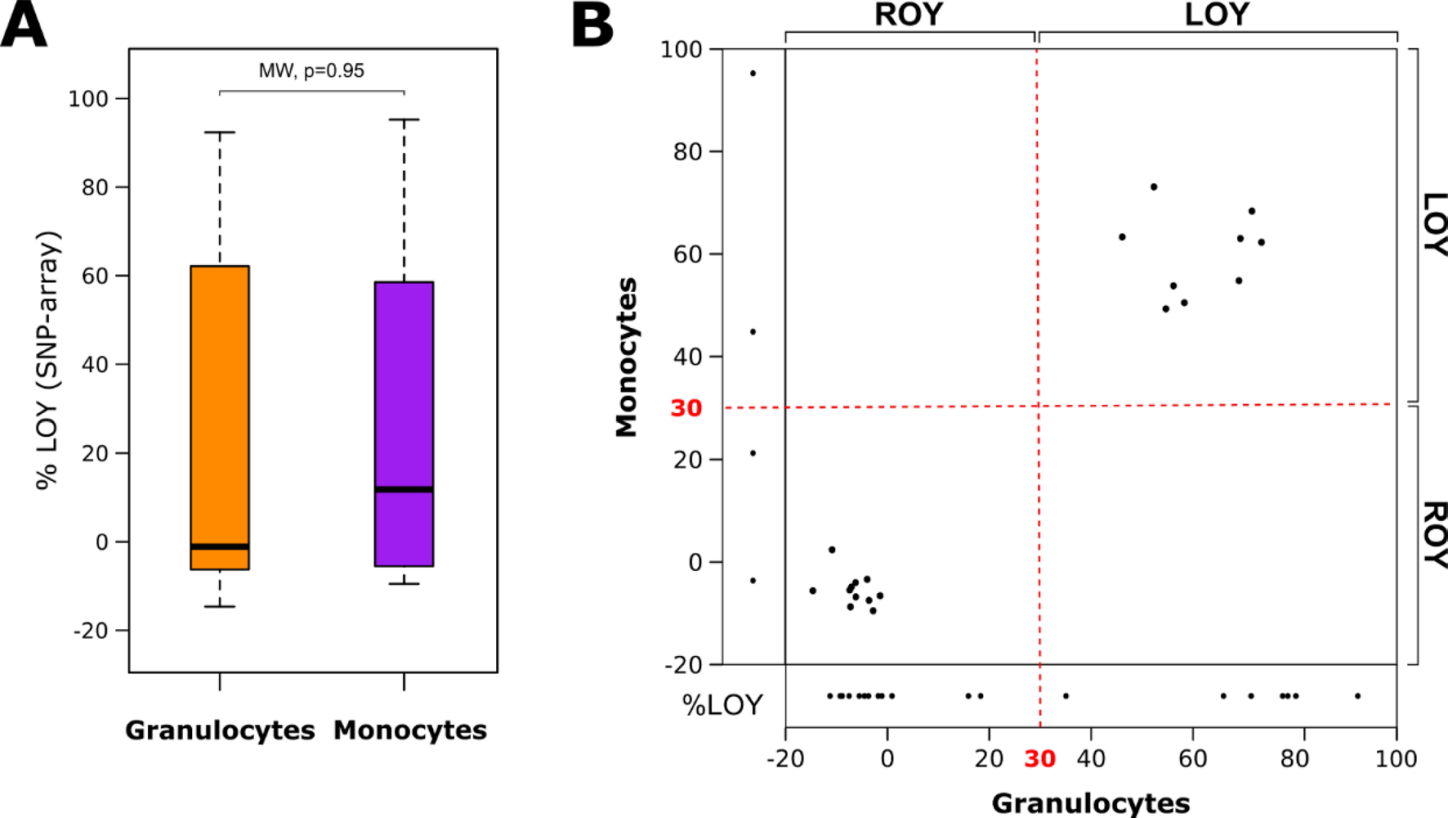
Distribution of LOY levels among the studied samples. **A** General distribution of %LOY cells among the two sampled cell types. P-value from Mann–Whitney (MW) pairwise test is present at the top, which is not significant, with no major differences in %LOY between granulocytes and monocytes. **B** Distribution of %LOY in granulocytes (X-axis) and monocytes (Y-axis). Every point in the main panel represents a measurement of %LOY for matched samples collected from the same patients. Narrow panels along each of the axes show results for LOY levels for unmatched samples in each cell type, due to insufficient DNA availability. The red dotted line denotes a 30% threshold used to divide the samples into LOY and ROY groups

In order to investigate the effect of LOY on CpG methylation in the context of AD, we analyzed blood samples from 43 men with LOAD (median age 78 years, age range 63–90 years) (Supplementary File 1—Table S1). The leukocytes were sorted using fluorescence-activated cell sorting (FACS) to obtain pure cell populations of granulocytes and monocytes. The samples studied were selected from previously reported individuals [4] and the only selection criterion was the availability of a sufficient amount of DNA, to perform CpG methylation profiling (Fig. 1). The status of LOY mosaicism for each cell type was assessed using SNP arrays, following a previously described method [1, 9]. The obtained mLRRY values (median Log R Ratio values of probes located in the male-specific part of chromosome Y) were transformed to estimate the percentage of LOY (%LOY) in each sample [6]. In total, LOY status was determined for 39 and 24 samples of granulocytes and monocytes, respectively (Fig. 2). We used a 30% cutoff (*i.e.,* 30% of the cells having LOY, as presented previously [6, 9] to divide the samples into “AD-LOY” and “AD-ROY” (the latter standing for Retention Of chromosome Y) groups (Supplementary File 1—Table S1). This revealed two separate clusters of data (Fig. 2B). Matched samples from granulocytes and monocytes (collected from the same patients) presented a high concordance of the %LOY estimates (Pearson correlation = 0.97, Fig. 2B).

### Genome-wide profiling of DNA methylation landscape

**Fig. 3:**
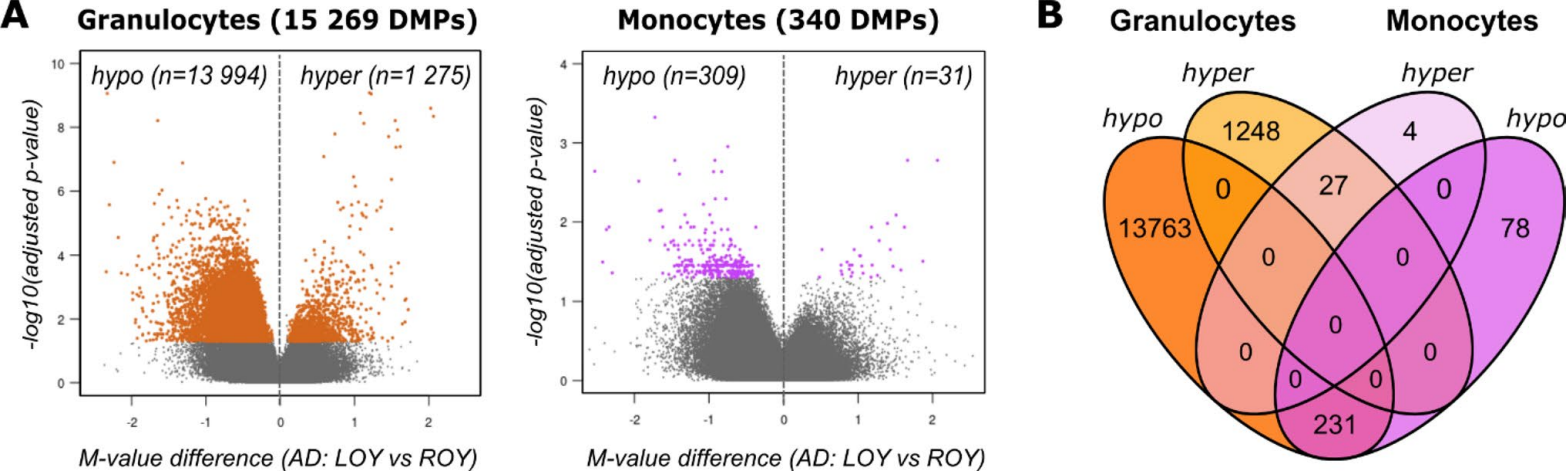
Results from analyses of differential methylation in LOY vs. ROY among AD patients. **A** Volcano plots of the genome-wide differential methylation analyses in granulocytes and monocytes. Black points denote all tested CpG sites. Colored dots represent the significant DMPs (FDR < 0.05, avDiff > 0.1). The direction of methylation change (X-axis) is shown in reference to the LOY samples (hypo—less methylated in cells with LOY, hyper—more methylated in cells with LOY). **B** Venn diagram comparing the number of hyper- and hypomethylated DMPs in granulocytes and monocytes identified by comparing LOY to ROY samples among AD patients

Using the methylation EPIC Beadchip we performed genome-wide DNA methylation analyses among the AD patients. Specifically, we obtained methylation data for 39 granulocyte and 24 monocyte samples covering 684,663 CpG sites, after dropping the problematic probes with known SNPs [58] and those related to smoking [59]. To identify LOY-related DNA methylation changes we performed a differential methylation analysis by comparing AD-LOY against AD-ROY groups. CpG probes located on the Y chromosome were also excluded from the analyses (see Methods). In granulocytes and monocytes, respectively, were identified 15,269 and 340 significantly differentially methylated probes (DMPs; adjusted p-value < 0.05 and absolute M-value difference > 0.1, Supplementary File 1—Tables S2 and S3). As shown in Fig. 3A, hypomethylation in AD-LOY samples dominated, both in granulocytes (13,994 of 15,269 DMPs, 92%) and monocytes (309 of 340 DMPs, 91%). Of note, 258 DMPs were shared between granulocytes and monocytes by comparing AD-LOY to AD-ROY samples among AD patients (p-value < 2.2e−16, hypergeometric test) (Fig. 3B). Investigation of the genomic distribution of the identified DMPs showed that they were mainly localized within the gene body, intergenic regions, and the so-called open sea regions (see Fig. S1).

### Gene-associated methylation changes

We next analyzed genes associated with the identified DMPs, *i.e.* differentially methylated genes (DMGs) between AD-LOY and AD-ROY groups (Supplementary Figs. S2A and B, Supplementary File 1—Tables S8 and S9). In total, we found 7,105 DMGs in granulocytes. Importantly, 4177 of these genes had at least one DMP within their promoters, defined as ± 2 kb from transcription start site, (TSS). The corresponding numbers for monocytes were 252 and 142 DMGs. Granulocytes and monocytes shared a total of 228 DMGs (Supplementary Fig. S2C).

Numerous DMGs (~ 43–45% in granulocytes and ~ 45–49% in monocytes) were associated with AD according to the OpenTargets [60] and GeneCards [61] databases, respectively (Supplementary File 1, Tables S9 and S10, Supplementary Figure S3)**.** We conducted a hypergeometric test and found that AD-associated genes were significantly enriched among DMGs, both using OpenTargets (granulocytes: p-value < 2.2e−16; monocytes: p-value = 3.0e−10) and GeneCards (granulocytes: p-value = 5.2e−05; monocytes: p-value = 0.019) databases of AD genes. Additionally, 179 DMGs in granulocytes and 8 DMGs in monocytes were previously found to exhibit LOY-associated dysregulation (Supplementary Fig. S3) [4].

### CpG methylation status is linked with changes in gene expression

**Fig. 4:**
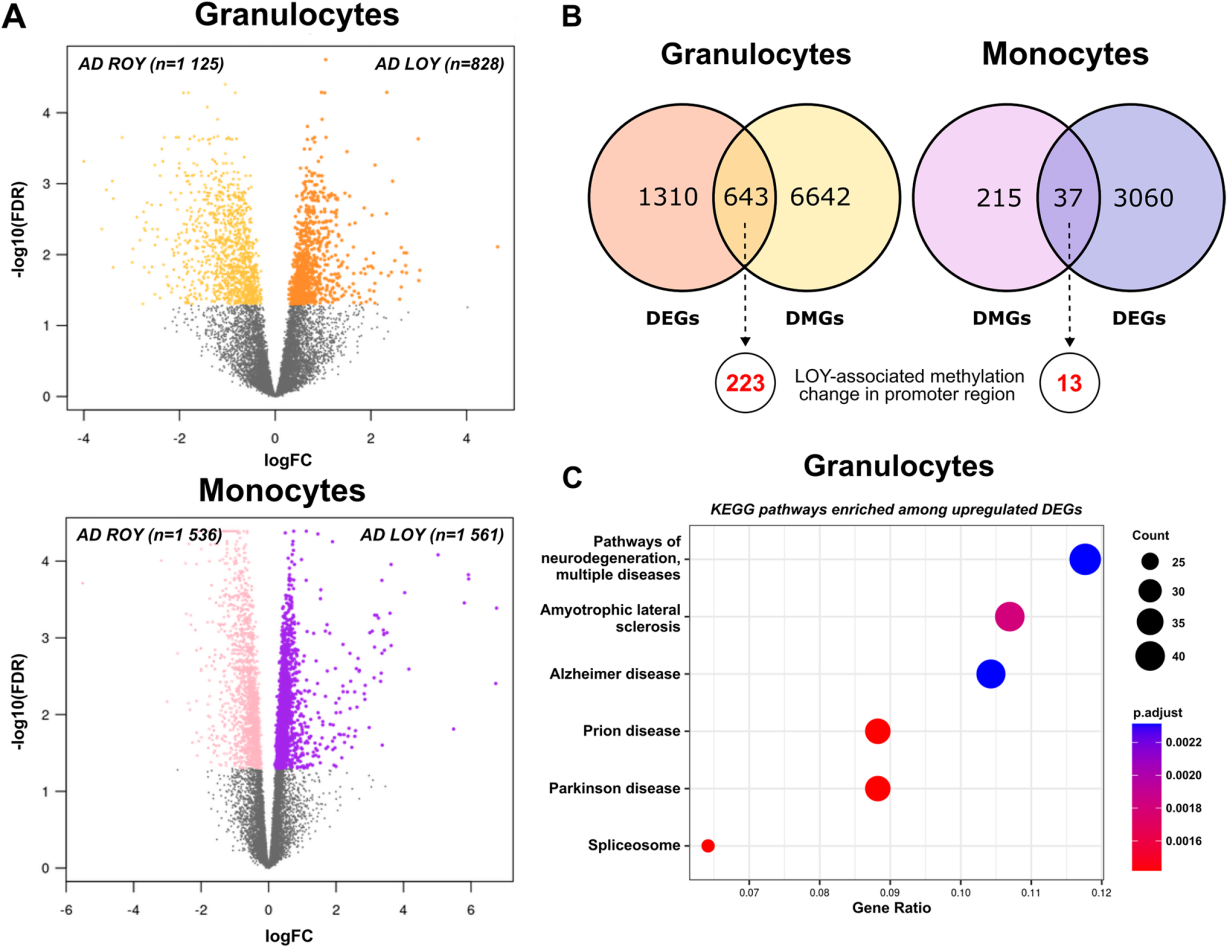
Results from differential expression analyses and the correspondence of promoter-related methylation with the observed expression patterns. **A** Volcano plots of the genome-wide differential expression analyses using bulk RNA-seq data from corresponding patients’ samples. Colored dots represent genes with significant change in expression (DEGs, FDR < 0.05). Direction of expression change (X-axis) refers to the LOY samples (up—higher in LOY, down—lower in LOY). **B** Venn diagrams comparing gene sets derived from the differential expression and differential methylation analyses. Red numbers in the circles below the diagram represent the genes with LOY-associated methylation change in promoter region. **C** Results of KEGG pathway enrichment analyses for DEGs upregulated in AD-LOY granulocytes. The size of the circle corresponds to the number of genes representing the given pathway. The color of the circle denotes the adjusted p-value from the enrichment analysis

To further explore the effects of DNA methylation changes, we reanalyzed RNA-seq data derived from Dumanski et al. [4]. Specifically, the analysis was conducted on available RNA from granulocyte and monocyte samples (31 AD individuals, see methods: Bulk RNA-seq data processing and reanalysis). We identified 1953 and 3097 genes demonstrating significant differential expression (DEGs) in granulocytes and monocytes, respectively (Fig. 4A, Supplementary File 1—Tables S4 and S5). While our differential methylation analyses showed that hypomethylated DMPs dominated, here the LOY-associated effect on gene expression in AD was less pronounced.

We further assessed to what extent DMGs and DEGs overlapped. In granulocytes, 643 genes were shared between the two analyses (p-value = 0.008739, hypergeometric test), and 223 of these had at least one LOY-associated DMP located within the promoter (p-value = 0.005688) (Fig. 4B, Supplementary File 1—Table S6). The corresponding numbers for monocytes were 37 (p-value = 0.5743) and 13 (p-value = 0.4040108) (Fig. 4B, Supplementary File 1—Table S7). Among granulocytes, DNA methylation of 380 (out of 545, 70%) probes were negatively correlated with the expression level of the associated gene (Supplementary File 1—Table S8), which is in line with the canonical model, where hypomethylation within CpG islands and promoter region is associated with increased gene expression [38]. Similarly, 13 DEGs in monocytes had differentially methylated sites within their promoters, and methylation changes of 11 of 15 probes (73%) were negatively correlated with the expression of the associated gene (Supplementary File 1—Table S8). We tested whether overlapping sets of DMGs and DEGs were enriched in any metabolic pathways. We found that genes upregulated in granulocytes were indeed enriched in KEGG pathways associated with neurodegeneration, Parkinson's disease, and AD (Fig. 4C). Detailed results of pathway enrichment analysis are shown in Supplementary File 1, Table S14.

### Examples of DMGs with LOY-associated transcriptional effect

**Fig. 5:**
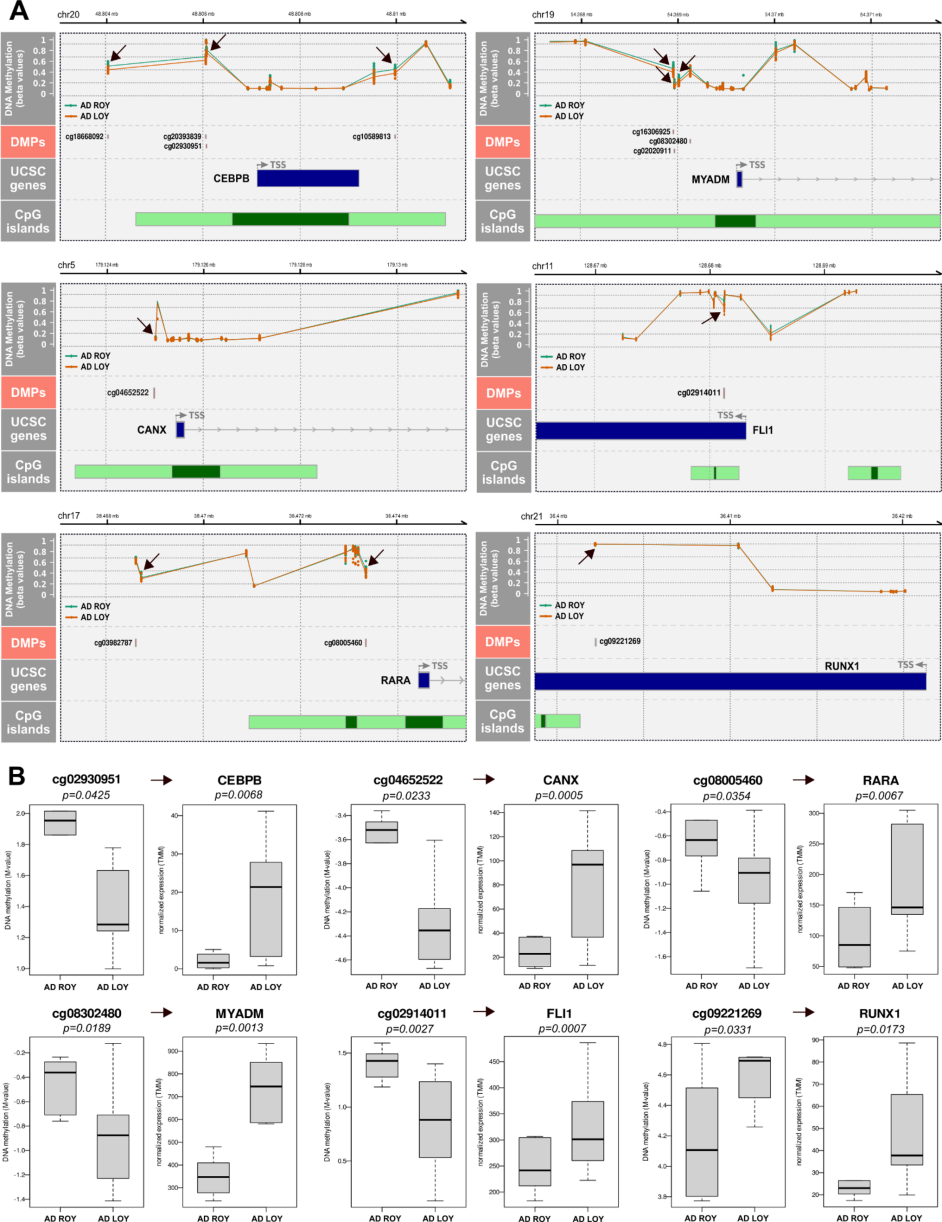
Genomic neighborhood of selected genes from granulocytes with changes in both DNA methylation and gene expression. **A** Genomic neighborhood of the promoter region of selected differentially expressed genes from granulocytes. All genes are upregulated in the LOY group in AD patients and have a hypomethylated site within their promoters. Top panel (DNA methylation) shows neighboring CpG probes located in the vicinity of the promoter region and their DNA methylation values in LOY (orange) and ROY (green) samples in AD. Black arrows point to differentially methylated probes. Second panel from top shows the identified significant differentially methylated sites. Panel named “UCSC genes” shows annotations of the gene’s structure. Blue rectangles show exons and introns are shown as thin lines. Arrows on introns show the direction of the gene from its 5’ side. TSS denotes the site of the start of transcription. The bottom-most panel shows CpG islands located in the vicinity of TSS. Dark green denotes CpG islands, and their shores are in light green. Chromosome name and the genomic coordinates are shown above each plot. **B** Promoter-associated CpGs with significant change in methylation in granulocytes juxtaposed with a relevant change in expression of the associated gene. Boxplots show methylation and expression values in one of the two compared groups—LOY or ROY in AD patients. Hypomethylation in LOY is associated with upregulation of the corresponding gene’s expression in LOY. BH-adjusted p-values are shown

We show selected, representative examples of genes from granulocyte analysis that follow the canonical model of methylation (Fig. 5). Additionally, according to the GeneCards database, all of these genes are highly related to AD. One of the genes meeting the canonical model is *CEBPB* (CCAAT enhancer binding protein beta), the expression of which was significantly upregulated in the AD-LOY group in granulocytes (logFC = 1.55, FDR = 0.043 (Supplementary Table S4). This one-exon gene harboring twelve CpG probes, had two statistically significant hypomethylated sites identified, both located upstream of its TSS (M-value difference 0.44–0.52, adjusted p-value = 0.008–0.043) (Fig. 5; Supplementary Table S2). Notably, C/EBP-β is an essential transcription factor during emergency granulopoiesis, and its level has been reported to be elevated in AD brain tissue compared to non-demented control subjects [62, 63]. Moreover, it has been shown that C/EBPβ is upregulated in hematopoietic stem/progenitor cells (HSPCs) under stress conditions in a mouse model [64]. Analogously, the *CANX* (Calnexin) gene illustrates the same pattern of DNA methylation and its gene expression in granulocytes. Our analysis showed that *CANX* was significantly upregulated in LOY granulocytes (logFC = 0.91, FDR = 0.008) and had a single hypomethylated site located within the promoter region (M-value difference 0.42, adjusted p-value = 0.023) (Fig. 5, Supplementary Table S2). Calnexin is involved in protein folding, functioning as a chaperone in the endoplasmic reticulum (ER) and this gene is upregulated in the frontal cortex of AD patients [65].

Another interesting example is the *RARA* gene (Retinoic Acid Receptor Alpha), which was upregulated in LOY cells in granulocytes (logFC = 0.74, FDR = 0.042) and contained a single promoter-related DMP (M-value difference 0.54, adjusted p-value = 0.035). Additionally, we detected four other DMPs localized outside of the promotor region of this gene, which were all hypomethylated as well (Fig. 5 and Supplementary File 1, Tables S2 and S6). A decline in the transcriptomic levels of retinoic acid receptors was suggested to be involved in the early stages of AD mouse models [66]. For the myeloid-associated differentiation marker gene *MYADM*, our analysis showed that three hypomethylated probes were identified in the promotor region of this gene (M-value difference 0.37–0.50, adjusted p-value = 0.0121–0.037). Also, *MYADM* was upregulated in LOY-harboring granulocytes (logFC = 0.622, FDR = 0.0153) (Fig. 5). This gene has been reported as upregulated during myeloid differentiation [67]. Genes previously associated with LOY, *FLI1* and *RUNX1* [68], which play a critical role in the regulation of hematopoiesis, were found to show statistically significant increase in expression in granulocytes with LOY (logFC = −0.62, FDR = 0.0007 for FLI1 and logFC = −0.64, FDR = 0.017 for RUNX1; Supplementary Table S4). However, their methylation patterns differed, namely, *FLI1* had a single hypomethylated site located within the promoter region (M-value difference 0.79, adjusted p-value = 0.00279; Supplementary Table S2), whereas, for *RUNX1*, our analysis showed that the DMP identified in the promoter region of this gene was hypermethylated (M-value difference 0.17, adjusted p-value = 0.0331; Fig. 5; Supplementary Table S2).

Among other genes exhibiting a canonical methylation pattern, specifically hypomethylation and increased expression (Supplementary file 1—Table S8), we identified those involved in oxidative stress (*OGG1*, *NFE2*) and apoptosis (*DFFB*, *TNFRSF1A*, *TRADD*). Notably, the canonical methylation pattern for *TRADD* was consistent between monocytes and granulocytes (Supplementary file 1—Table S8).

### Genes with LOY-associated changes in DNA methylation and expression are part of a large interaction network

**Fig. 6:**
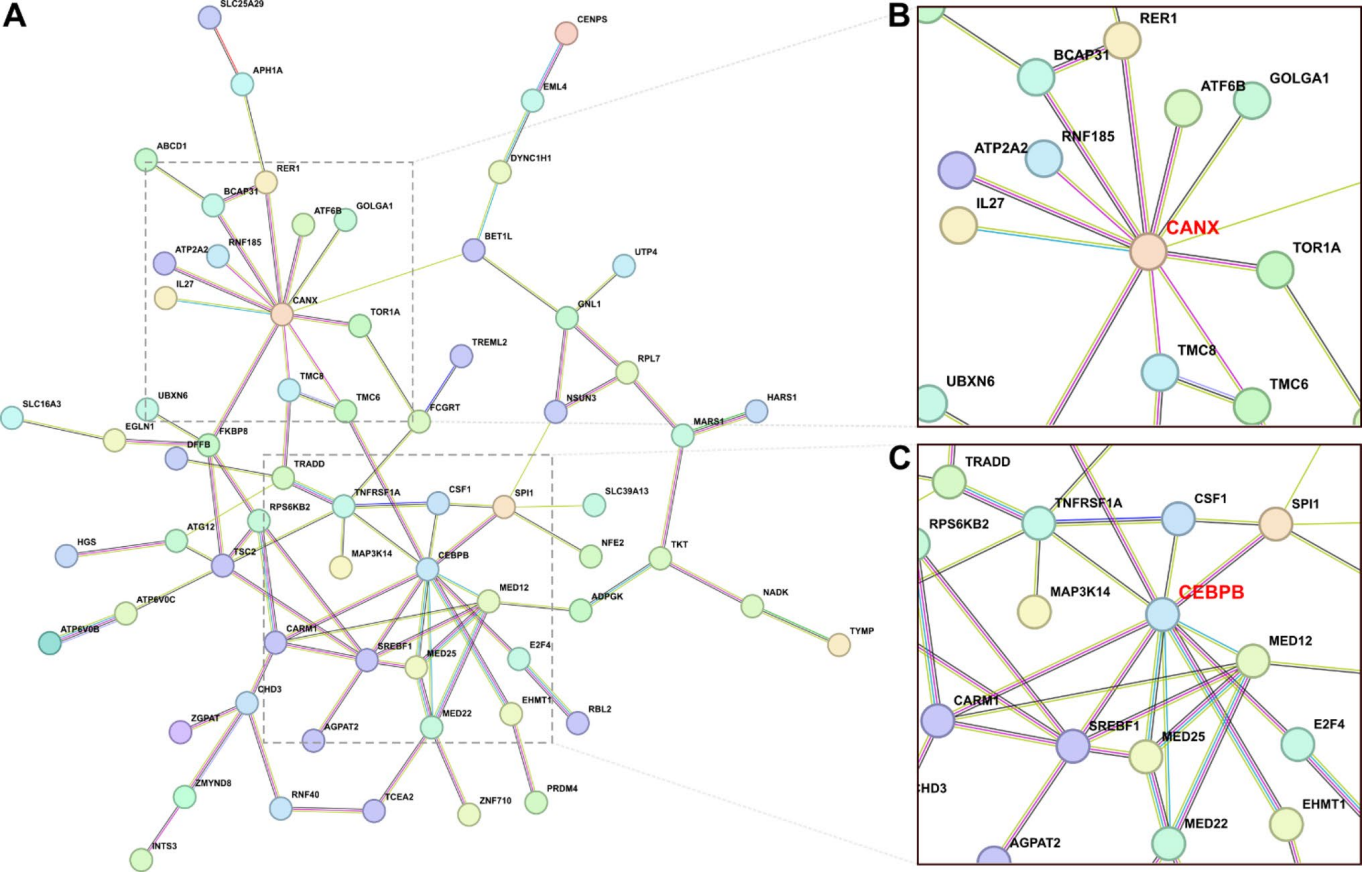
String protein–protein interaction network based on genes that display both variations in DNA methylation (within promoter region) and gene expression. Lines represent different types of evidence used in predicting the associations: the presence of fusion evidence (red), neighborhood (green), cooccurrence (blue), experimental (purple), text mining (yellow), database (light blue), coexpression (black). **A** 153 genes with changes in granulocytes were used as input for the interaction analyses (see Material and Methods). Edges represent known or predicted protein–protein interactions, e.g. from curated databases and experiments. **B** Selected hub protein encoded by the *CANX* gene (calnexin, calcium-binding protein), which has 12 known interactions with other products of DMG/DEG genes from granulocytes. **C** Selected hub protein encoded by the *CEBPB* gene (CCAAT Enhancer Binding Protein Beta), which has 11 known interactions with other products of DMG/DEG genes from granulocytes

In order to characterize the interaction between genes that exhibited variability in both DNA methylation and gene expression, we performed protein–protein interaction (PPI) analysis using the STRING database (see Methods). The resulting PPI network in granulocytes consisted of 105 edges and 153 nodes, representing 79 known and 7 predicted protein–protein interactions (Fig. 6A, Supplementary File 1—Table S11). We found two hub proteins with the highest number of connections encoded by the *CANX* and *CEBPB* genes, as the representatives of two important interaction networks (Fig. 6B and 6C, respectively). Specifically, the *CANX* (calnexin, calcium-binding protein) gene showed 12 known interactions with other products of DMG/DEG genes from granulocytes, while the *CEBPB* (CCAAT enhancer binding protein beta) gene had 11 known interactions with other products of DMG/DEG genes from granulocytes. No network could be identified for the gene set from monocytes.

### Regulating the regulators—LOY-associated effect on transcription factor binding

We used the promoter sequences of the identified DMG/DEG genes to determine if they possess any enriched transcription factor binding sites (TFBS) as compared to genes without significant change in DNA methylation between LOY and ROY groups (see Methods). In granulocytes, we found 190 enriched motifs (153 among hypomethylated genes, 37 for hypermethylated ones; Supplementary File 1—Table S12). In monocytes, 72 motifs were enriched among promoters of the studied genes (59 for hypomethylated and 13 for hypermethylated genes; Supplementary File 1—Table S13).

Among the top-ranking motifs in hypomethylated genes, both in granulocytes and monocytes, were those belonging to the SP transcription factors (SP1, SP2, SP3, and SP4). In granulocytes, the SP2 motif was ~ 1.6 times more frequent in promoters of hypomethylated DMG/DEG genes, compared to the background set of genes (FDR 2.91e−165). Interestingly, SP2 transcription factor itself was identified among the genes with a significant change in methylation and expression in granulocytes (hypomethylated, upregulated). Another interesting example was the E2F4 transcription factor, whose motif was enriched among hypermethylated genes in granulocytes.

## Discussion

Knowledge accumulated over the past decade on LOY suggests a causative effect of this aneuploidy on the pathogenesis of AD. Age is also the key factor associated with both LOAD and LOY in men. Despite that the two phenotypes are age-dependent, and the fact that LOY is strongly and independently associated with LOAD, the molecular mechanisms underlying the role of LOY in LOAD are not well studied. Furthermore, little is known about the potential LOY-induced methylation effects that may underlie the observed LOY-associated transcriptome changes [4]. For that reason, we have studied sorted subsets of leukocytes derived from male LOAD patients for changes of CpG-methylation and corresponding effects on the gene expression. As the microglia in brain and monocytes in blood circulation represent functionally related cells [31–34, 69], the rationale of our study was to focus on myeloid cell lineage from AD patients to elucidate the role of LOY in AD. Moreover, granulocytes and monocytes have been shown to be affected by LOY in AD patients, as shown in our previous study [4]. The samples studied here contained either high levels of mosaic LOY (> = 30% of cells) or were predominantly not affected by LOY, thus forming two groups used for comparisons. Moreover, we have related the changes in the CpG methylation with RNA analysis, which were performed in the same samples for many patients, allowing identification of up- and down-regulated genes, presumably as a consequence of change in the methylation state.

Granulocytes and monocytes from AD patients showed a similar percentage of LOY cells (Fig. 2A), which is consistent with frequent clonal expansions of LOY-cells in the myeloid lineage [4, 7]. By analyzing DNA methylation patterns in 39 granulocyte and 24 monocyte samples, we show herein a predominant hypomethylation in LOY cells in both studied subpopulations of cells. The overall number of significant differentially methylated genes (DMGs) was higher in granulocytes than in monocytes (15269 vs 340 DMGs, Fig. 3A). Noteworthy is that the majority of hypomethylated genes in monocytes (231 out of 309) were shared with hypomethylated genes found in granulocytes. This implies that the presumed LOY-induced methylation effects could have a core of defined targets regardless of the cell type. In contrast to predominant hypomethylation, gene expression analysis showed that the ratio between of up and down regulated genes is comparable in granulocytes and monocytes (Fig. 4A). Interestingly, 1095 significant differentially expressed genes were shared between granulocytes and monocytes. Of these, 474 were downregulated and 620 were upregulated. Only one gene, *CLEC16A,* showed a different direction of change as it was upregulated in monocytes and downregulated in granulocytes with LOY (Supplementary File 1—Tables S4 and S5). This large overlap of genes with the same direction of change in expression supports hypothesis that LOY could have gene-specific impact on transcriptome in myeloid and perhaps other types of cells.

An important finding from the combined analyses of methylation and gene expression is the dysregulation of DEGs, presumably caused by methylation changes. We identified 643 genes in granulocytes and 37 genes in monocytes, which had significantly altered methylation and expression patterns (Fig. 4B). According to the canonical model, hypomethylation within CpG islands and promoters is associated with increased expression, whereas hypermethylation results in diminished expression of the corresponding gene [38, 39]. In the comparison of LOY vs. ROY cells, we identified numerous clear cases of DMG and DEG pairs fitting the canonical model (Fig. 5, Supplementary File 1—Table S8). A good example of a previously reported hypomethylated gene in the blood of LOAD patients is *B3GALT4* (Beta-1,3-Galactosyltransferase 4) [42]. Importantly, this gene was upregulated and hypomethylated in our analysis of granulocytes with LOY, which together may indicate that the effect of hypomethylation can be further exacerbated by LOY. Another interesting candidate gene linking differential methylation, LOY and male health is the *RARA* gene, which is hypomethylated and upregulated in LOY cells. The *RARA* gene, encoding retinoic acid receptor alpha (RARα) that binds retinoic acid (RA), activates a signaling cascade leading to the transition of promyelocytes into the mature white blood cells through the granulocytic line [70, 71]. Low levels of ligand result in upregulation of *RARA* expression and consequently lead to inhibition of the differentiation of mature myeloid cells. High expression of *RARA* can also influence leukocyte differentiation through its interaction with C/EBPβ [72]. C/EBPβ is an essential transcription factor during emergency granulopoiesis, an immunological response induced by pathogens, including viral disease [62]. It should also be mentioned that independent GWAS study identified SNP variant in the *C/EBPβ* gene is among the loci that predispose to LOY [2]. Moreover, it was shown that posttranslational modifications of C/EBP-β can influence lymphoid to myeloid cell trans-differentiation [73]. Our finding that both genes important for leukocyte differentiation (*RARA* and *C/EBPβ*) are hypomethylated and upregulated in LOY cells implies that LOY might significantly impact on the maturation of myeloid lineage. Intriguingly, our independent studies have revealed that LOY appears abundantly in cells of the first line of immune defense, i.e. the innate immune response [4, 7]. These cells are mainly monocytes and neutrophils, with the latter constituting the largest population of granulocytes and having the second-highest rate of daily cell turnover [74]. Thus, LOY may drive selective maturation pathways in myeloid cells through epigenetic mechanisms regulating key differentiation regulators like *RARA* and *CEBPB*. Another example of a myeloid differentiation factor gene found to be hypomethylated and upregulated in our analysis is *MYADM*, which exhibits augmented expression during myeloid cell formation [67, 75]. Therefore, our results might be converted into a marker for immature hematopoietic cells committed toward myelopoiesis.

Our analysis also revealed that granulocytes with LOY show higher expression of *FLI1* and *RUNX1*. Both genes are differentially methylated in LOY cells with *FLI1* being hypomethylated and *RUNX1* hypermethylated. FLI1 is a transcription factor, whose higher expression favors the differentiation into megakaryocytes rather than erythroid cells [68]. FLI1 interacts with RUNX1 and GATA2, which play a key role in the development of the hematopoietic system. Notably, *RUNX1* expression is following the upregulation of *FLI1* [76]. Independent genome-wide association studies have shown that regions enriched in LOY heritability overlapped with binding sites of various transcription factors, among them FLI1, RUNX1, and GATA2 [68]. In our study, we detected LOY-related hypomethylation of the NF-E2 gene, which is another transcription factor functioning within the FLI1 network [77]. It has been shown that the triad of transcriptional factors, NF-E2, FLI1, and RUNX1 cooperate in regions of dynamic chromatin in the late-differentiation stage of megakaryocytes in a mouse model [78]. Excessive expression of NF-E2 in CD34 + causes the expansion of erythroid progenitor cells, a consequence of delays in the early phase of erythroid maturation [79]. Furthermore, overexpression of *NF-E2* in a murine myeloproliferative neoplasm model causes several hematopoietic phenotypes, such as leukocytosis and excessive thrombocytosis, along with a chronic inflammation creating an oxidative stress environment [80]. Notably, increased production of reactive oxygen species is related to excessive activation of neutrophils, which is observed in AD patients [37, 81]. It has been observed that high levels of reactive oxygen species (ROS) lead to rapid accumulation of 8-oxoG in DNA, which triggers cellular responses and among them, increased expression of *OGG1* [82]. This factor regulates the expression of inflammatory cytokine-encoding genes such as CCL20, IL-1B, TNFa, CXCL1, or CXCL2 [83, 84]. The molecular mechanism is based on the binding of OGG1 to 8-oxoGs located in inflammation-related genes facilitating the binding of NFkB transcription factor and their expression [85]. Considering the above, the hypomethylation of the *OGG1* gene detected in our analysis, manifested by its increased expression could lead to an exacerbated inflammatory response of the immune system.

Another noteworthy aspect is that leukocytes with LOY derived from AD patients may possibly exhibit apoptosis-related processes. Indeed, our analysis revealed that genes involved in the regulation of apoptosis, in particular *TRADD*, *TNFRSF1A*, and *DFFB*, were differentially methylated. TRADD, the TNF receptor-related death domain, through interactions with the intracellular death domain (DD), binds to activated TNFRSF1A (tumor necrosis factor receptor superfamily member 1A), leading to programmed death and NF-κB activation [86]. TNFRSF1A, expressed in neutrophils, plays a key role in TNFα-adapted apoptosis, and its blocking has anti-apoptotic effects [87]. It has been observed that proapoptotic TRADD was upregulated at the transition from myelocytes/metamyelocytes (MYs) to mature neutrophils in humans [88]. Additionally, DFFB which codes caspase-dependent DNase, triggers apoptosis through DNA fragmentation and chromatin condensation.

LOY at a single-cell level is a binary event removing the entire chromosome, resulting in loss of ~ 2% of the haploid genome [89], which likely influences the packaging of DNA and chromosomes within a nucleus. Specifically, *KDM5D* and *UTY*/*KDM6C* have histone demethylase activity and loss of these functions due to LOY could have a profound impact on epigenetic regulation in leukocytes. For instance, KDM5D targets H3K4me3 of histones [90] and this chromatin landmark is usually found near TSSs, which is an indicator of transcriptionally active genes. Thus, in the event of LOY, we could expect changes at the level of gene expression, triggered by chromatin remodeling. Recent studies showed dysregulation of epigenetic processes in AD and that regulation may occur through chromatin higher-order structures [47, 48, 91]. In this context, we should also mention that global hypomethylation of the genome, which we report here for normal granulocytes and monocytes with LOY, is frequent in tumors and it has been linked to genomic instability of tumor cells [92, 93]. This possible effect of LOY and its interplay with the chromatin remodeling via complete deletion of *KDM5D* and *UTY/KDM6C* genes deserves further studies.

Our study presents an analysis of the effect of LOY on epigenetic modifications in granulocytes and monocytes from AD patients. While previous research has consistently demonstrated an association between LOY and AD [4, 9, 15, 30] compared to non-demented individuals, our study does not provide a control group. This is because our primary objective was the assessment of LOY and ROY cells in sorted leukocytes, wherein ROY cells served as a control. In conclusion, we provide further evidence suggesting that LOY in immune cells plays a role in the pathogenesis of LOAD in men. Our combined analyses of CpG methylation as well as expression analysis identified new candidate genes and we confirm numerous genes already implicated in the pathogenesis of LOAD. The results are also well aligned with the hypothesis that age-related dysfunction of the immune system cells is one of the major factors contributing to the development of AD. LOY is also reflected in higher-level epigenetic changes that show an AD-specific pattern, further contributing as a potential biomarker of the disease.

## Supplementary Information

Below is the link to the electronic supplementary material.

Supplementary file1 (XLSX 5475 KB)

Supplementary file2 (DOCX 302 KB)

## Data Availability

The DNA methylation data used in this study are available from the authors upon a reasonable request. The bulk RNA-seq datasets are available upon a reasonable request from the authors of the original publication [4].

## Abbreviations

LOY: Loss of chromosome Y
ROY: Retention of chromosome Y
AD: Alzheimer’s disease
DMP: Differentially methylated probe
DEG: Differentially expressed gene
DMG: Differentially methylated gene
TSS: Transcription start site
LOAD: Late onset Alzheimer’s disease
EOAD: Early onset Alzheimer’s disease
RNA-seq: Bulk RNA sequencing
PBMCs: Peripheral blood mononuclear cells
mLRRY: Median Log R Ratio values of probes from the male-specific part of chromosome Y
FACS: Fluorescence-activated cell sorting

## Code availability

Unless otherwise stated, all the data processing and visualization of the results was done in R (version 3.6.3). The code used is available from https://github.com/jakalssj3/LOY-AD-meth
