# Supplementary material for: DNA methylation patterns contribute to changes of cellular differentiation pathways in leukocytes with LOY from patients with Alzheimer´s disease
Source: Cell Mol Life Sci. 2025 Feb 25;82(1):93. doi: 10.1007/s00018-025-05618-8 (PMC11861481; doi:10.1007/s00018-025-05618-8)
Supplement: Supplementary file 2 — Supplementary file2 (DOCX 302 KB) [file 18_2025_5618_MOESM2_ESM.docx]

**SUPPLEMENTARY FIGURES**

**Figure S1**


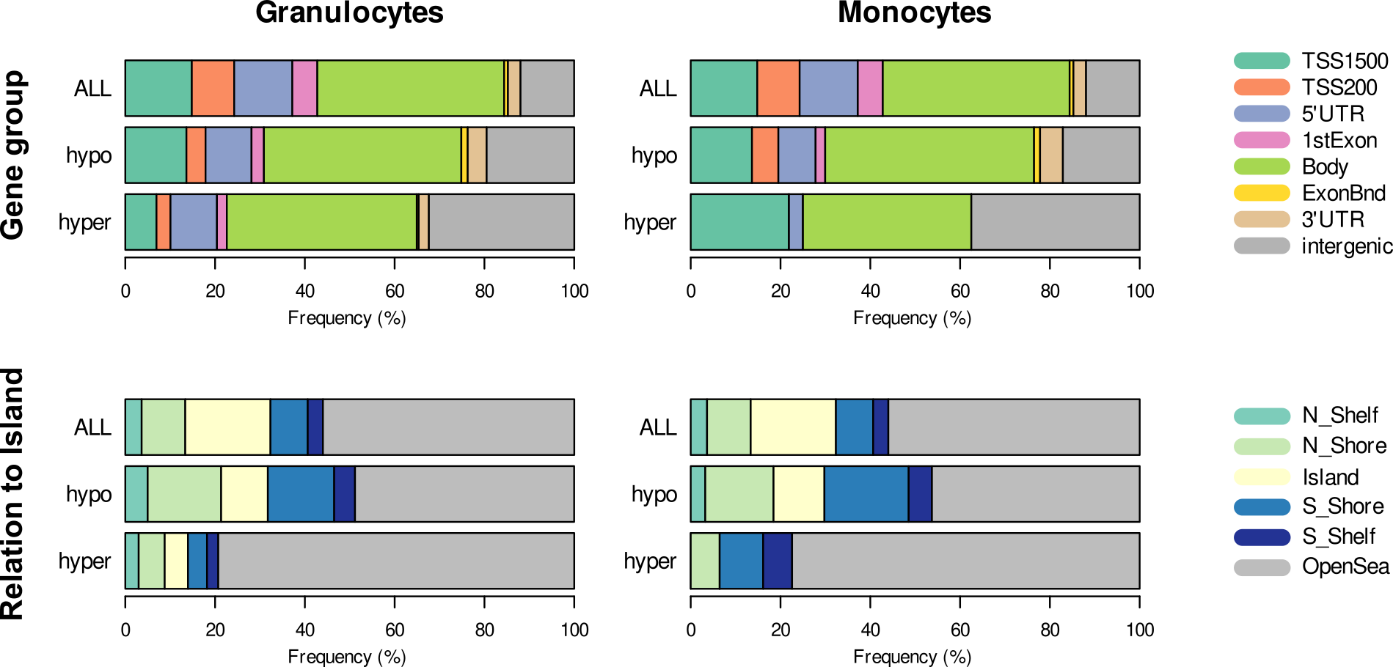


**Figure S1.** Genomic distribution of the identified hyper- and hypomethylated DMPs as compared to the distribution of all probes on the EPIC chip. **Top panel.** Distribution of probes and DMPs in relation to gene annotations (UCSC_RefGene_Group column in the Infinium MethylationEPIC Manifest). The left and right panels correspond to granulocytes and monocytes, respectively. **Bottom panel.** Distribution of probes and DMPs in relation to CpG islands (Relation_to_Island column in the Infinium MethylationEPIC Manifest).

**Figure S2**


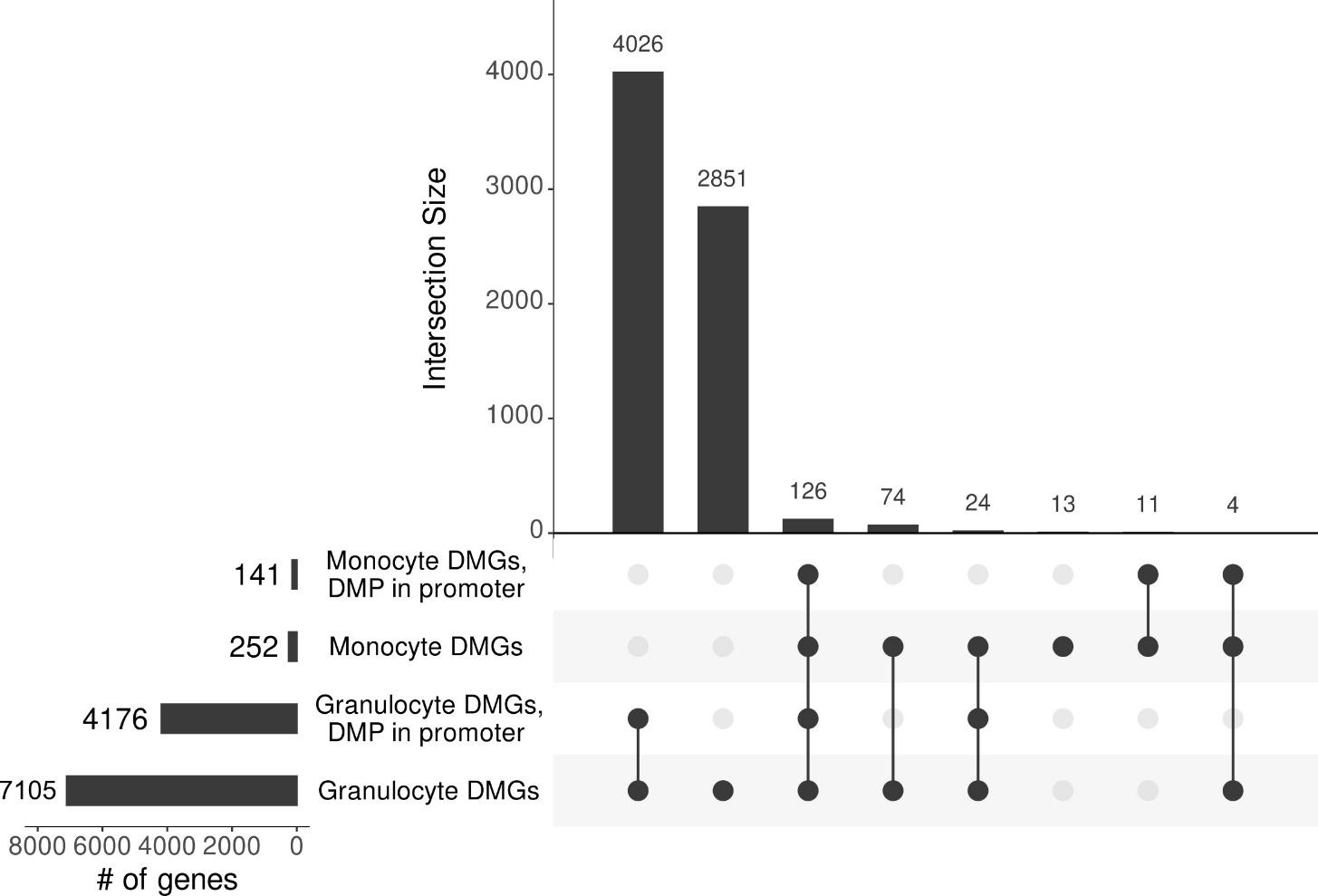


**Figure S2.** Upset plot showing intersections of genes with significantly differentially methylated CpGs identified in monocytes and granulocytes. Names of the sets of genes are mentioned beside the bars. Vertical lines refer to genes shared between the sets.

**Figure S3**


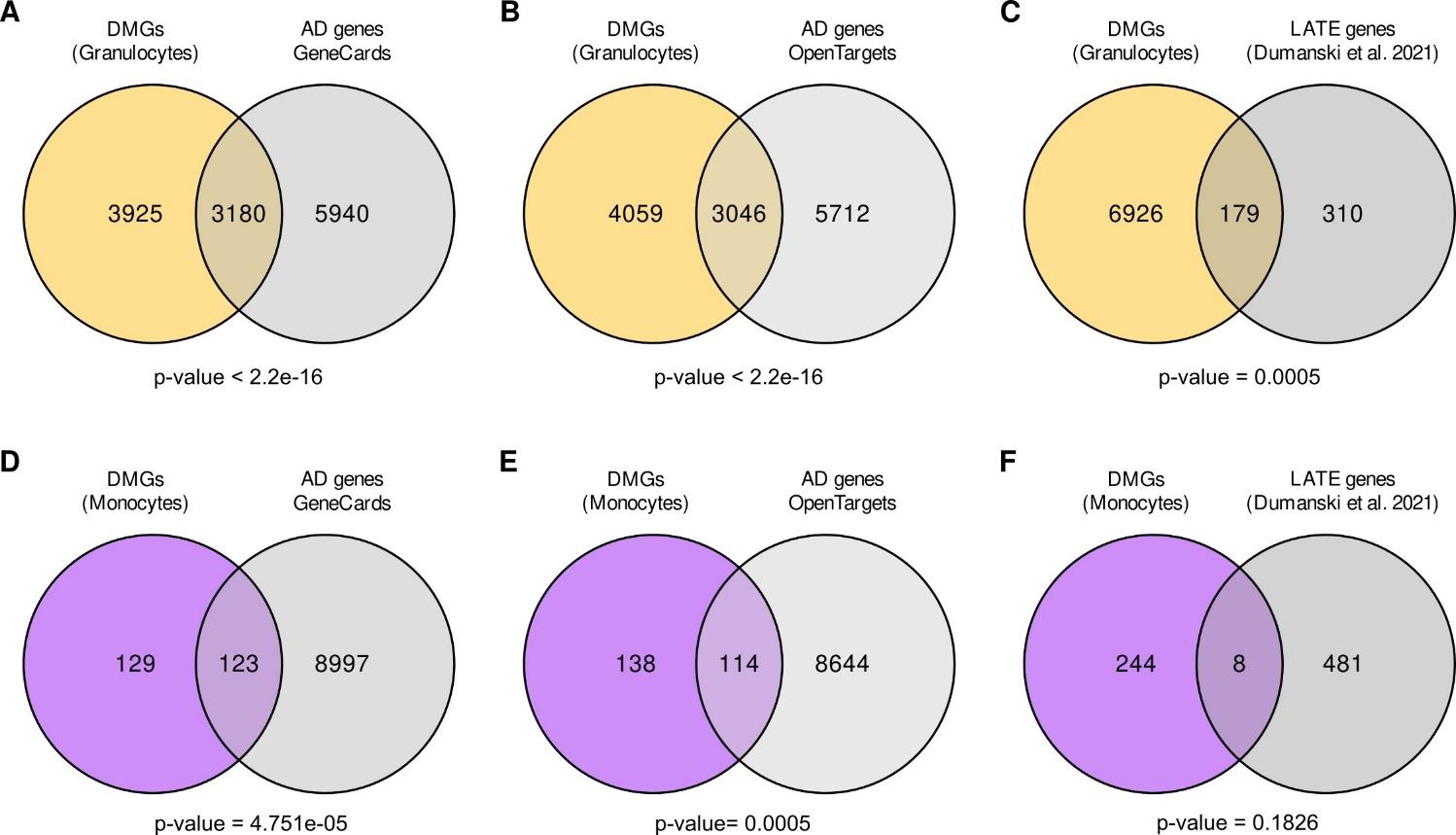


**Figure S3.** Intersection of genes containing differentially methylated probes (DMGs) with sets of genes identified as related to Alzheimer’s disease. (**A**) Intersection of granulocyte DMGs with AD-related genes from GeneCards database (**B**) Intersection of granulocyte DMGs with AD-related genes from OpenTargets database (**C**) Intersection of granulocyte DMGs with so-called LATE genes from Dumanski et. al 2021 (**D**) Intersection of monocyte DMGs with AD-related genes from GeneCards database (**E**) Intersection of monocyte DMGs with AD-related genes from OpenTargets database (**F**) Intersection of monocyte DMGs with LATE genes.
